# Mutations in *PMR1* stimulate xylose isomerase activity and anaerobic growth on xylose of engineered *Saccharomyces cerevisiae* by influencing manganese homeostasis

**DOI:** 10.1038/srep46155

**Published:** 2017-04-12

**Authors:** Maarten D. Verhoeven, Misun Lee, Lycka Kamoen, Marcel van den Broek, Dick B. Janssen, Jean-Marc G. Daran, Antonius J. A. van Maris, Jack T. Pronk

**Affiliations:** 1 Department of Biotechnology, Delft University of Technology, Van der Maasweg 9, 2629 HZ Delft, The Netherlands; 2 Department of Biochemistry, Groningen Biomolecular Sciences and Biotechnology Institute (GBB), University of Groningen, Nijenborgh 4, 9747 AG Groningen, The Netherlands

## Abstract

Combined overexpression of xylulokinase, pentose-phosphate-pathway enzymes and a heterologous xylose isomerase (XI) is required but insufficient for anaerobic growth of *Saccharomyces cerevisiae* on d-xylose. Single-step Cas9-assisted implementation of these modifications yielded a yeast strain expressing *Piromyces* XI that showed fast aerobic growth on d-xylose. However, anaerobic growth required a 12-day adaptation period. Xylose-adapted cultures carried mutations in *PMR1*, encoding a Golgi Ca^2+^/Mn^2+^ ATPase. Deleting *PMR1* in the parental XI-expressing strain enabled instantaneous anaerobic growth on d-xylose. In *pmr1* strains, intracellular Mn^2+^ concentrations were much higher than in the parental strain. XI activity assays in cell extracts and reconstitution experiments with purified XI apoenzyme showed superior enzyme kinetics with Mn^2+^ relative to other divalent metal ions. This study indicates engineering of metal homeostasis as a relevant approach for optimization of metabolic pathways involving metal-dependent enzymes. Specifically, it identifies metal interactions of heterologous XIs as an underexplored aspect of engineering xylose metabolism in yeast.

In conventional feedstocks for fermentative production of fuel ethanol, such as corn starch and cane sugar, carbohydrates predominantly occur as dimers or polymers of hexose sugars. These hexose sugars can be efficiently and rapidly fermented by *Saccharomyces cerevisiae*. Economically feasible ethanol production from non-food lignocellulosic feedstocks additionally requires efficient, anaerobic fermentation of d-xylose and l-arabinose^1^,^2^. Although wild-type *S. cerevisiae* strains cannot ferment these pentose sugars, they can slowly convert d-xylulose^3^. In yeast species that can grow on d-xylose, such as *Scheffersomyces stipitis*, its metabolism is initiated by a two-step conversion into d-xylulose by the combined activity of a xylose reductase (XR) and a xylitol dehydrogenase (XDH)^4^. The different redox cofactor preferences of XR and XDH represent a challenge in their use for constructing d-xylose-fermenting *S. cerevisiae* strains. This redox problem causes the production of substantial amounts of xylitol by anaerobic cultures of such engineered strains^5^,^6^,^7^. Elegant engineering strategies in which cofactor specificities of XR and/or XDH were altered, have not yet completely eliminated the formation of this by-product^8^,^9^.

In bacteria, d-xylose conversion is often initiated by its direct isomerization to d-xylulose, catalysed by xylose isomerase (XI) (EC 5.3.1.5). Until 2003, attempts to express heterologous XI genes in *S. cerevisiae* yielded no or very low XI activities under physiologically relevant conditions^10^,^11^,^12^,^13^. Then, multi-copy expression of a newly discovered xylose isomerase gene (*xylA*) from the anaerobic fungus *Piromyces* sp. E2^14^ was shown to yield high XI activity in cell extracts of *S. cerevisiae*^15^,^16^. *Piromyces xylA* shows strong sequence similarity with *Bacteroides* XI genes, suggesting that the fungus acquired the gene by horizontal gene transfer. Indeed, expression of XI genes from *Bacteroides* species also yielded XI activity in *S. cerevisiae*^17^,^18^.

Consistent with the slow growth of wild-type *S. cerevisiae* strains on d-xylulose^3^, functional expression of *xylA* by itself only enabled very slow aerobic growth on xylose^15^,^16^. Kuyper *et al*. (2005a)^19^ reported that expression of *xylA* combined with constitutive overexpression of the genes encoding the native *S. cerevisiae* xylulokinase (*XKS1*, EC 2.7.1.17), ribulose 5-phosphate epimerase (*RPE1*, EC 5.3.1.1), ribulose 5-phosphate isomerase (*RKI1*, EC 5.3.1.6), transketolase (*TKL1*, EC 2.2.1.1) and transaldolase (*TAL1*, EC 2.2.1.2) was sufficient to enable anaerobic growth on d-xylose, at a specific growth rate of 0.07 h^−1^. Several subsequent studies confirmed that overexpression of a heterologous XI, combined with overexpression of xylulokinase and the enzymes of the non-oxidative pentose-phosphate pathway, is required for fast anaerobic fermentation of d-xylose^20^,^21^).

Laboratory evolution experiments designed to further improve the kinetics of xylose fermentation revealed expression of the heterologous XI as a key factor, as reflected by amplification of the XI gene via formation of extra-chromosomal circular DNA^22^ or increased numbers of XI genes on the yeast chromosomes^21^. Other studies demonstrated improved xylose fermentation in yeast strains in which XI expression was increased by random mutagenesis, codon optimization or by mutations influencing protein folding^23^,^24^,^25^. Additional mutations that improve pentose-fermentation kinetics, mainly identified by resequencing of laboratory-evolved strains, affected structural genes encoding native yeast hexose transporters^26^,^27^,^28^,^29^,^30^ and in the ‘secondary’ transaldolase and transketolase isoenzymes *NQM1* and *TKL2*^20^,^31^.

Over a decade of intensive research on d-xylose fermentation by XI-based, engineered *S. cerevisiae* strains yielded many important insights into their physiology. However, one important and industrially relevant aspect remains incompletely understood. While an initial study^19^ reported that combined overexpression of *xylA*, xylulokinase and non-oxidative pentose-phosphate pathway enzymes was sufficient to enable anaerobic growth of *S. cerevisiae* on d-xylose, subsequent reports indicated that anaerobic growth on xylose required additional, as yet unidentified mutations^21^,^23^.

The aim of the present study was to investigate the molecular basis for anaerobic growth of engineered *xylA*-expressing, d-xylose-metabolizing *S. cerevisiae*. To this end, we used CRISPR-Cas9 mediated genome editing for single-step construction of an *S. cerevisiae* strain that grew aerobically on d-xylose as sole carbon source. After adaptation to anaerobic growth in xylose-grown bioreactor batch cultures, we showed that mutations in a single gene enabled anaerobic growth on xylose. Via a combination of physiological and enzymological analyses, we investigated how these mutations affected intracellular metal homeostasis and d-xylose metabolism.

## Results

### One-step construction of a xylose-utilizing *Saccharomyces cerevisiae* strain

**Figure 1:**
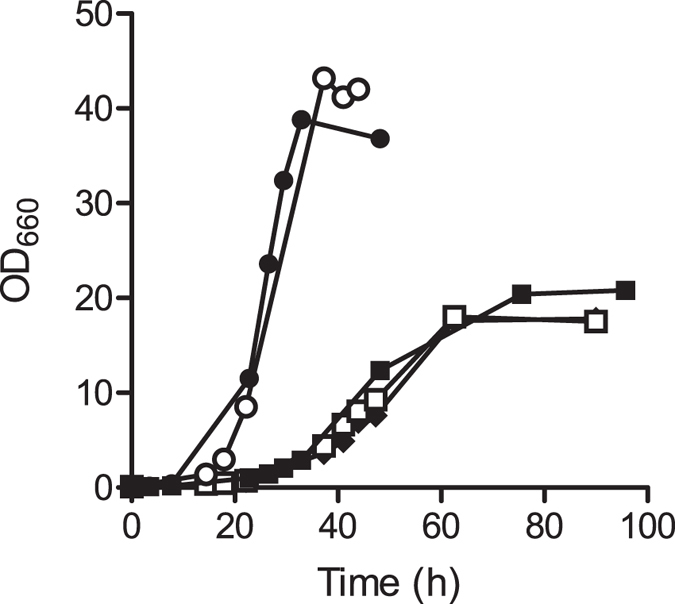
Growth of *S. cerevisiae* strains with different *PMR1* alleles in aerobic cultures on xylose. Aerobic growth curves in shake-flask cultures grown on synthetic medium with 20 g l^−1^ xylose. Symbols indicate the following *S. cerevisiae* strains: ●, IMX696 (*xylA*, PPP↑, *XKS1*↑), ■, IMX906 (*xylA*, PPP↑, XKS1↑, *pmr1*Δ), ○, IMX979 (*xylA*, PPP↑, XKS1↑, *PMR1*),◆, IMS0488 (isolate from IMX696 culture adapted to anaerobic growth on xylose carrying Pmr1^G249V^ mutation) and □, IMS0489 (isolate from IMX696 culture adapted to anaerobic growth on xylose carrying Pmr1^W387*^ mutation). Data shown are from a single flask experiment for each strain. For all strains, data obtained from independent duplicate experiments differed by less than 5%.

To construct a xylose-metabolizing *S. cerevisiae* strain, nine copies of an expression cassette containing *Piromyces xylA*, as well as single expression cassettes for constitutive overexpression of the native yeast genes for xylulokinase (*XKS1*) and for the enzymes of the non-oxidative branch of the pentose-phosphate pathway (*RKI1, RPE1, TKL1, TKL2* and *TAL1*) were introduced in *S. cerevisiae* CEN.PK113-7D. Additionally, an expression cassette for *NQM1,* a paralog of *TAL1* whose duplication has been shown to enhance pentose fermentation by engineered *S. cerevisiae*^31^, was introduced. Combination of *in vivo* assembly^32^ and CRISPR/Cas9-mediated chromosomal integration^33^ enabled a one-step introduction of all expression cassettes in the *GRE3* locus, thereby inactivating *GRE3*, which encodes a non-specific aldose reductase that can reduce xylose to xylitol^34^. The nine copies of the *xylA* cassette were introduced as tandem repeats to facilitate adaptation of the *xylA* copy number by homologous recombination. Transformants obtained after plating on xylose synthetic medium (SMX) plates were restreaked thrice on the same medium. The genome of the resulting strain IMX696 (Table 1), in which correct integration of the cassettes was confirmed by diagnostic PCR using primers listed in Table S3, was sequenced to assess whether mutations had occurred during growth on SMX plates. No single-nucleotide polymorphisms (SNPs), insertion/deletions in coding regions or changes in chromosomal copy numbers were observed. However, read-depth analysis revealed the presence of 36 rather than 9 copies of the *xylA* cassette. This amplification of *xylA* is consistent with earlier reports that showed a positive impact of high *xylA* copy numbers on xylose metabolism by engineered *S. cerevisiae* strains^21^,^22^,^25^. In aerobic shake-flask cultures on SMX, strain IMX696 exhibited a specific growth rate of 0.21 h^−1^ (Fig. 1).

### Anaerobic growth on xylose requires prolonged adaptation

**Figure 2:**
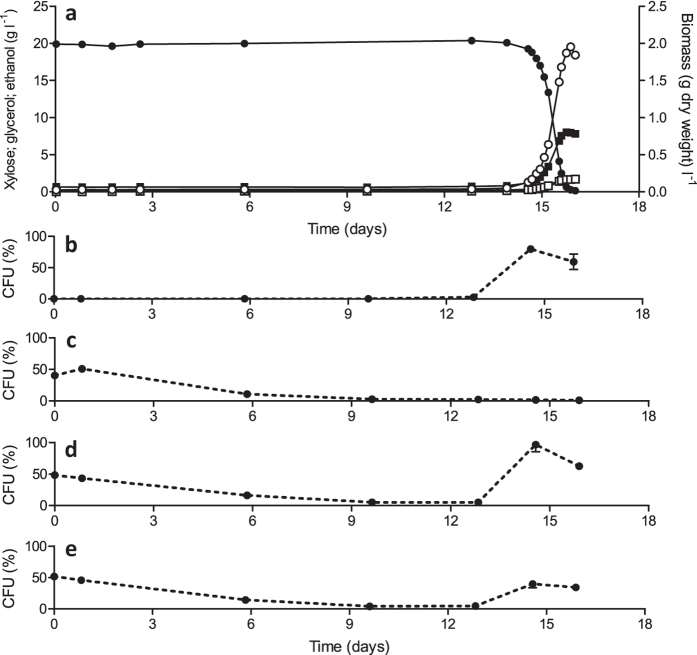
Anaerobic growth of *S. cerevisiae* IMX696 (*xylA*, PPP↑, *XKS1*↑) on xylose requires a prolonged adaptation period. (**a**) Growth, xylose consumption and product formation after inoculation of aerobically pregrown cells in anaerobic bioreactors containing synthetic medium with xylose (20 g l^−1^). Symbols: ●, xylose, ■, ethanol, ○, biomass, □, glycerol. (**b**) Colonyforming units (CFU) on anaerobically incubated xylose medium reflect adaptation to growth on xylose in the absence of oxygen. (**c**) CFU on aerobically incubated xylose medium reflect trade-off between aerobic and anaerobic growth on xylose. (**d**) and (**e**) CFU on anaerobically and aerobically incubated glucose medium, respectively, showing that oxygen sensitivity of cells adapted to anaerobic growth on xylose is not carbon-source dependent. Data shown in this figure are from one of two independent replicates, the replicate experiment is shown in Supplementary Fig. S2.

Anaerobic growth of the engineered xylose-fermenting strain IMX696 was investigated in nitrogen-sparged bioreactor cultures on SMX, supplemented with the anaerobic growth factors Tween-80 and ergosterol. In duplicate experiments, CO_2_ production, which was continuously monitored in the off-gas of the bioreactors, was only observed after 12 days of incubation (Supplementary Fig. S1). To investigate this slow adaptation to anaerobic growth on xylose in more detail, the experiment was repeated, with regular analysis of culture viability, metabolite concentrations and growth (Fig. 2). Again, no significant xylose consumption occurred during the first 12 days of the experiment. A subsequent increase in biomass concentration coincided with the conversion of xylose to ethanol and glycerol. The specific growth rate after the onset of anaerobic growth was estimated at 0.11 h^−1^ based on biomass dry weight measurements during the mid-exponential growth phase. Biomass and ethanol yields on xylose were 0.086 ± 0.01 g biomass (g xylose)^−1^ and 0.382 ± 0.01 g ethanol (g xylose)^−1^, respectively (Fig. 2a, Supplementary Fig. S2a). The dynamics of adaptation to anaerobic growth were further investigated by plating culture samples on synthetic medium with either glucose (SMD) or xylose (SMX). Colony counts on these plates were determined after aerobic and anaerobic incubation (Fig. 2b–e). On anaerobic SMX plates, colonies were first observed after 10 d, at which time they represented a fraction of only 1.8∙10^−4^ of the number of cells that were plated. Subsequently, consistent with the exponential growth observed by biomass dry weight measurements, the fraction of cells capable of anaerobic growth of xylose rapidly increased (Fig. 2b). When culture samples were plated on SMD, aerobic and anaerobic plates showed similar trends in colony counts (Fig. 2d,e). Conversely, plating on SMX revealed a strong trade-off between the ability to grow aerobically and anaerobically on xylose. On aerobically incubated SMX plates cell counts did not increase, not even when exponential growth on xylose took off during the final days of the bioreactor experiments and strongly increasing colony counts were observed on anaerobically incubated SMX plates (Fig. 2c).

### Adaptation to anaerobic growth on xylose coincides with mutations in *PMR1*

The dynamics of colony counts on SMX plates (Fig. 2b) suggested that adaptation to anaerobic growth might have involved one or more mutations. To test this hypothesis, the genomes of strains IMS0488 and IMS0489, which were isolated from the independent anaerobic adaptation experiments shown in Fig. 2 and Supplementary Fig. S2, were sequenced. Read-depth analysis of both strains revealed a decrease of the *xylA* copy number to 24 and 25, respectively, as compared to 36 in the parental strain IMX696. No other changes in chromosomal copy numbers were observed. Strikingly, both strains carried non-synonymous SNPs in the coding region of *PMR1* (Table 2), which encodes a high-affinity Golgi Ca^2+^/Mn^2+^ P-type ATPase^35^. These mutations caused a single amino acid change (Pmr1^G249V^) in strain IMS0488 and introduced a premature stop codon (Pmr1^W387*^) in strain IMS0489.

### Mutations in *PMR1* enable anaerobic growth on xylose

**Figure 3:**
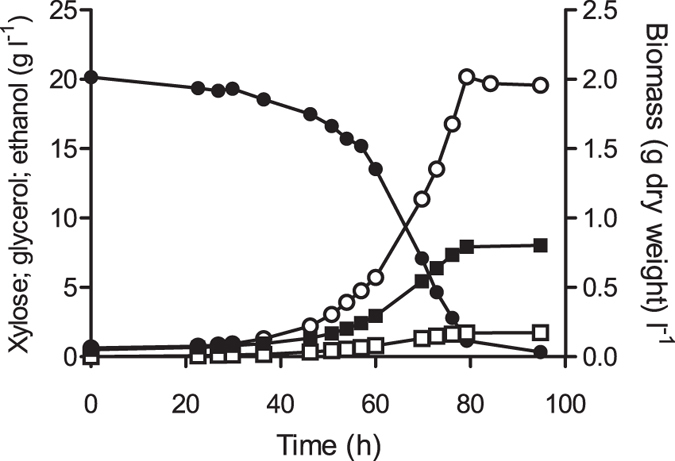
Deletion of *PMR1* enables anaerobic growth on xylose of engineered *S. cerevisiae* without prior adaptation phase. Growth and product formation of *S. cerevisiae* strain IMX906 (*xylA*, PPP↑, *XKS1*↑, *pmr1*Δ on xylose (20 g l^−1^) in anaerobic bioreactors. Symbols: ●, xylose, ■, ethanol, ○, biomass, □, glycerol. The data shown are from one of two independent replicates.

To investigate the role of the *PMR1* mutations in the adaptation to anaerobic growth on xylose, the gene was deleted in the parental strain IMX696. In replicate anaerobic bioreactor cultures on xylose, the resulting strain IMX906 grew within 24 h and completely consumed all sugar within 70 h (Fig. 3). The specific growth rate of both cultures was 0.08 h^−1^, while biomass and ethanol yields on xylose were 0.086 g ± 0.01 biomass (g xylose)^−1^ and 0.40 g ± 0.01 ethanol (g xylose)^−1^, respectively. To further investigate the role of the *PMR1* deletion in the instantaneous anaerobic growth of strain IMX906 on xylose, the wild-type *PMR1* allele was reintegrated in this strain. The resulting strain IMX979 showed a lag phase of over 250 h in duplicate anaerobic bioreactor cultures on xylose (Supplementary Fig. S1), thereby confirming the key role of *PMR1* inactivation in the ability of engineered, XylA-based *S. cerevisiae* to grow anaerobically on xylose.

The plate count experiments during the anaerobic adaptation phase on xylose suggested a trade-off between aerobic and anaerobic growth on xylose (Fig. 2b,c). This possible trade-off was further explored by growth experiments in aerobic shake flasks on SMX. In these experiments, strains in which *PMR1* was mutated or deleted consistently showed a lower specific growth rate than strains that carried a wild-type *PMR1* allele (0.10 h^−1^ and 0.21 h^−1^, respectively; Fig. 1). Furthermore, aerobic xylose-grown shake-flask cultures of strains with mutated *PMR1* alleles accumulated ethanol to 3–4-fold higher concentrations than corresponding cultures of strains with wild-type *PMR1* alleles (Supplementary Fig. S3). Consistent with previous results^36^, aerobic shake flask cultures growing on glucose also revealed an approximately 50% reduced growth rate of the *pmr1Δ* strain IMK692 (Supplementary Fig. S4).

### Mutations in *PMR1* affect intracellular metal concentrations in xylose-metabolizing *S. cerevisiae* strains

Pmr1 is an ATP-dependent transporter that imports Ca^2+^ and Mn^2+^ into the Golgi complex^37^. Based on the observation that *pmr1* null mutants accumulate Ca^2+^ and Mn^2+^ intracellularly, Pmr1 has also been implicated in secretion of divalent metal ions via the Golgi complex^38^. To explore a possible relation between metal homeostasis and anaerobic growth on xylose, we analysed intracellular concentrations of Ca^2+^, Mn^2+^, Mg^2+^ and Fe^2+^ in biomass samples from anaerobic mid-exponential phase bioreactor cultures using inductively coupled plasma mass spectrometry. Contents of Mg^2+^, Ca^2+^ and Fe^2+^ were similar in all analysed strains, with Mg^2+^ accounting for over 80% of the analysed divalent metal ions, followed by Ca^2+^, and with Fe^2+^ accounting for less than 1% of the measured metals. Conversely, large differences were observed for the Mn^2+^ content. While in strains with a wild-type *PMR1* allele, Mn^2+^ represented less than 0.2% of the measured metal ions, 12- to 29-fold higher Mn^2+^ contents were observed in strains with mutated *PMR1* alleles, irrespective of whether they were grown on xylose or glucose (Table 3). The observation that mutations in *PMR1* affected cellular contents of Mn^2+^ but not those of Ca^2+^ is consistent with a previous study^39^.

### Activity and metal content of xylose isomerase expressed in *S. cerevisiae* strains

Laboratory evolution studies have identified XI activity as a key factor in rapid fermentation of xylose to ethanol^16^,^21^,^22^. XI enzymes are known to be metal dependent, with pronounced differences in metal binding and impact of metal identity on enzyme kinetics^40^. To examine the impact of Mn^2+^ on XylA activity, XI activities were assayed in cell extracts of strains IMX906 (*pmr1Δ*) and its parental strain IMX696 after aerobic and anaerobic growth on SMD in shake-flask cultures (Supplementary Fig. S5). Cell extracts from both strains exhibited similar activities in assays without added metal ions. These activities do not necessarily reflect *in vivo* metal binding as they may, for example, have been influenced by binding of metals released during preparation of cell extracts, e.g. by disruption of vacuoles. Addition of Mn^2+^ and, to a lesser extent, of Mg^2+^ to the XI assays yielded significantly higher XI activities than observed in the absence of added metals. Conversely, addition of Ca^2+^ led to lower activities.

For a further analysis of the effect of Mn^2+^ on XylA activity, we purified the enzyme from the controlled anaerobic bioreactor cultures that were also used to determine cellular metal contents (Supplementary Fig. S6). Concentrations of Mg^2+^, Ca^2+^, Fe^2+^ and Mn^2+^ were measured in purified protein samples and the amount of each metal per enzyme active site was calculated (Table 4). These analyses showed that the isolated enzymes contained fewer than two metal ions per subunit, indicating that their metal binding sites were not fully occupied. In independent replicate experiments, large and consistent differences were observed in the Mn^2+^ contents of XylA isolated from strains with wild-type and mutated *PMR1* alleles (0.017 and 0.30 mol Mn (mol XylA subunit)^−1^, respectively, Table 4). The higher Mn^2+^ content of XylA isolated from xylose- or glucose-grown cells of the *pmr1Δ* strain coincided with a ca. 2-fold higher specific activity than measured with enzyme purified from the *PMR1* strain (Table 4). Although metal binding may have changed during cell disruption and enzyme purification, this correlation does indicate that Mn^2+^-loaded XylA is a better catalyst than the Mg^2+^-loaded enzyme. Addition of 1 mM MgCl_2_ to purified enzyme preparations enhanced their XI activities, consistent with incomplete metal loading in the cell and/or metal loss during purification and activity assays.

### Mn^2+^ binding results in superior catalytic efficiency of XylA

To accurately analyse the effect of different metals on catalytic properties of XylA, apoenzyme was prepared from XylA isolated from xylose-grown cultures of strain IMX906. Subsequently, XI activities were measured after reconstitution of apo-XylA with Mg^2+^, Ca^2+^ or Mn^2+^ (Table 5). The activities of Mn^2+^- and Mg^2+^ -reconstituted apo-XylA were higher than activities in non-metal-supplemented assays with XylA purified from yeast cultures (Table 4). The reconstituted enzyme showed the highest catalytic efficiency in the presence of Mn^2+^, with a *k*_cat_/*K*_M_ ratio that was 4-fold and 1500-fold higher than with Mg^2+^ and Ca^2+^, respectively. Both the highest *k*_cat_ and the lowest *K*_M_ were observed with Mn^2+^ and contribute to the superior catalytic efficiency with this metal cofactor (Table 5). When XylA apoenzyme was reconstituted with mixtures of divalent metals that resembled those observed in intracellular metal content analyses (Table 3) of strains IMX696 (*PMR1*) and IMX906 (*pmr1Δ*), an 80–90% increase of XI activity was observed as the fraction of Mn^2+^ was increased from 0.002 to 0.01 (Supplementary Table S1).

## Discussion

One-step, Cas9-assisted integration of a heterologous XI (*Piromyces* XylA) and overexpression of native yeast genes encoding xylulokinase and enzymes of the non-oxidative pentose-phosphate pathway (PPP) enabled fast aerobic growth on xylose by *S. cerevisiae*, thus illustrating the efficiency of Cas9-based genome editing in this yeast^33^,^41^. Nine copies of the *xylA* cassette were incorporated in tandem to facilitate expansion or compression of the *xylA* copy number by homologous recombination. This approach was validated by the four-fold higher *xylA* copy number in transformants isolated on xylose medium and its decrease in independent replicate cultures after subsequent adaptation to anaerobic growth. The observed amplification of *xylA* was consistent with the previously reported positive impact of high *xylA* copy numbers on xylose metabolism^20^,^21^,^22^. In line with earlier studies^21^,^23^, this metabolic engineering strategy did not enable anaerobic growth on xylose. In principle, the engineered XI-based pathway should allow for efficient, redox-cofactor-balanced alcoholic fermentation on this sugar. However, anaerobic growth on xylose requires much higher fluxes through XI since the ATP yield of anaerobic, fermentative metabolism of this sugar is approximately eight-fold lower than that of its aerobic, respiratory dissimilation (assuming an *in vivo* P/O ratio of 1.0)^42^.

In independent replicate cultures, anaerobic growth on xylose required a two-week adaptation, which was shown to reflect the accumulation of spontaneous mutants with single-nucleotide mutations in *PMR1*. The observation that single, easily acquired point mutations enabled this adaptation may explain an earlier report that overexpression of XylA, xylulokinase and PPP enzymes sufficed to enable anaerobic growth of *S. cerevisiae* on xylose^19^. Here, we demonstrate that inactivation of *PMR1* caused both a strongly elevated intracellular Mn^2+^ concentration and an increased loading of heterologously expressed XylA with Mn^2+^. Moreover, *in vitro* studies showed that loading of XylA apoenzyme with Mn^2+^ led to higher enzyme activities than binding of other divalent metal ions present in the yeast cytosol.

Consistent with the conclusion that intracellular Mn^2+^ homeostasis affects anaerobic xylose metabolism through its impact on *in vivo* XylA activity, none of the five *S. cerevisiae* enzymes that subsequently convert d-xylulose into glycolytic intermediates (xylulokinase, ribulose-5-phosphate isomerase, ribulose-5-phosphate 3- epimerase, transaldolase and transketolase) have been documented to be Mn^2+^ dependent (BRENDA database^43^). Our results do not exclude the possibility that altered Mn^2+^ levels influenced pentose metabolism by mechanisms other than influencing XylA activity. However, a key role of XylA is consistent with the observation that acquisition of mutations in *PMR1* coincided with a decrease of the *xylA* copy number from 36 to 25. This decrease, which occurred during the course of a single batch culture, suggests that mutations in *PMR1* may have affected a trade-off between the need for a high *in vivo* activity of XylA and the metabolic burden associated with its high-level synthesis. As demonstrated in a study on the energetic impacts of galactose-induced synthesis of the enzymes of the Leloir pathway^44^, such a metabolic burden is much more pronounced in anaerobic cultures than in aerobic, respiring cultures due to the lower ATP yield from fermentative sugar dissimilation.

The mutations in *PMR1* that enabled anaerobic growth on xylose negatively affected aerobic growth. High intracellular Mn^2+^ concentrations have previously been implicated in impaired mitochondrial function^36^, which is consistent with the increased accumulation of ethanol in aerobic shake-flask cultures of *pmr1* strains (Supplementary Fig. 3). Moreover, TORC1 signalling, which is involved in regulation of mitochondrial respiratory functions, is inhibited by Mn^2+^ and Pmr1 has been identified as a negative regulator of *TOR1*, which encodes a subunit of the TORC1 complex^45^. Additionally, Mn^2+^-induced apoptosis mediated by Ndi1^46^,^47^, a mitochondrial NADH dehydrogenase, may have contributed to low colony counts observed when cultures adapted to anaerobic growth on xylose were plated under aerobic conditions (Fig. 1c). The reduced growth rate in aerobic cultures of strains carrying *PMR1* mutations should be considered when their anaerobic industrial application is preceded by an aerobic biomass propagation phase.

Despite the pivotal role of the functional expression of a heterologous XI in *S. cerevisiae*^18^ and the well documented role of metal ions in the active sites of XIs from taxonomically diverse organisms^48^, the impact of metal loading on the performance of heterologously expressed XIs in *S. cerevisiae* has previously not been investigated. Similar to bacterial XIs^40^, apo-XylA isolated from yeast could be activated with different metals. The results of this study suggest that metal loading can have a large effect on the *in vivo* catalytic performance of the enzyme.

The pronounced influence of cellular metal content on XI activity was in agreement with its promiscuity towards metal cofactors found in the *in vitro* analyses. However, while the fraction of the XI-bound Mn^2+^ increased by more than 10-fold in strains that carried mutations in *PMR1*, cellular contents of Mn^2+^ were still at least 40 times lower than the combined Mg^2+^ and Ca^2+^ contents (Tables 3 and 4). This observation suggests that the affinity of XylA for Mn^2+^ is higher than for the other divalent metal ions. Functional expression of heterologous XIs in *S. cerevisiae* initially represented a formidable challenge in engineering *S. cerevisiae* for anaerobic xylose fermentation^10^,^11^,^12^,^13^. After *Piromyces* *xyla*^14^, XI genes from several eukaryotes and prokaryotes sources were shown to also be functionally expressed in *S. cerevisiae*, including those from *Orpinomyces* sp^49^, *Arabidopsis thaliana*^50^, *Clostridium phytofermentans*^23^, *Bacteroides thetaiotaomicron*^18^, *Bacteroides stercoris, Prevotella ruminicula TC2-24*^51^ and *Sorangium cellosum*^52^. In view of the key catalytic role of metal ions in all known xylose isomerases, we expect that *in vivo* activity of these and other XIs in yeast cells will also be affected by engineering of metal homeostasis.

Mutations in *PMR1* have been identified in two previous studies on adaptive laboratory evolution of XylA-based, engineered *S. cerevisiae* strains. Klaassen *et al*.^53^ identified a mutation in *PMR1* (Y38C) in a strain evolved for fermentation of l-arabinose and xylose to ethanol. Recently, Hou *et al*.^25^, reported a mutation in *PMR1* (G698V) in a respiratory-deficient XylA based *S. cerevisiae* strain obtained by adaptive laboratory evolution on xylose medium. Our results strongly suggest that, in both studies, the mutations in *PMR1* may have contributed to the selected phenotypes. Additionally, the superior catalytic efficiency of Mn^2+^-loaded XylA may explain a recent report that MnSO_4_ supplementation enhanced growth on xylose of acetate-stressed cultures of a XylA-based xylose-fermenting *S. cerevisiae* strain^54^.

Our study demonstrates the importance of metal homeostasis and enzyme loading in XI-based yeast metabolic engineering strategies for anaerobic conversion of xylose-containing lignocellulosic feedstocks into fuels and chemicals. Inactivation of *PMR1*, combined with overexpression of PPP enzymes, xylulokinase and *xylA* was shown to be sufficient to enable anaerobic growth of *S. cerevisiae* on xylose. Beyond xylose utilization, engineering of metal homeostasis has the potential to improve *in vivo* performance of other metal-dependent heterologous enzymes or pathways.

## Methods

### Strains and maintenance

All *S. cerevisiae* strains used in this study (Table 1) originate from the CEN.PK lineage^55^,^56^. Frozen stock cultures were stored at −80 °C in 30% (vol/vol) glycerol.

### Plasmid and strain construction

Plasmids used in this study are presented in Supplementary Table S2. Expression cassettes required for xylose fermentation were introduced into the *GRE3* locus of *S. cerevisiae* strain IMX581 by simultaneous *in-vivo* assembly and integration^32^. Expression cassettes for *RPE1, RKI1, TAL1, NQM1, TKL1, TKL2* and *XKS1* were obtained by fusing constitutive promoter sequences, ORFs and terminator sequences amplified from CEN.PK113-7D in a fusion-PCR^57^ using the primers specified in Supplementary Table S3. Plasmid pYM-N18^58^ was used as a template for the *TEF1* promoter. The resulting fragments were cloned into pJET-1.2 blunt-end vectors. Correct assembly was verified by sequencing as described below. PCR amplification of expression cassettes and plasmids was performed using Phusion Hot Start II High Fidelity DNA Polymerase (Thermo Scientific, Waltham, MA), according to the manufacturer’s protocol. Integration in *GRE3* locus was mediated by a chimeric CRISPR/Cas9 editing system^33^,^41^ with gRNA expressed from an episomal plasmid^33^. The plasmid backbone was PCR amplified from pMEL10 using primers 5792–5980 (Supplementary Table S3). A plasmid insert containing the 20 bp gRNA-targeting sequence was obtained by PCR amplification with primers 5978–5979 using pMEL10 as template. The resulting fragment was fused to the plasmid backbone with the Gibson Assembly Cloning kit (New England Biolabs, Ipswich, MA), yielding plasmid pUDE335. *E. coli* DH5a cells were transformed with 1 μL of the Gibson-assembly mix using a Gene PulserXcell Electroporation System (Biorad, Hercules, CA). Plasmid DNA was isolated from *E. coli* cultures using a Sigma GenElute Plasmid kit (Sigma-Aldrich, St. Louis, MO). The presence of the *GRE3* cutting gRNA was confirmed by PCR-amplification using primer pair 2528–960 followed by digestion with FastDigest *ClaI* (Thermo Scientific).

The coding region of the *Piromyces* sp. E2 xylose isomerase gene [Genbank: CAB76571.1] was codon optimized according to the codon preference of highly expressed glycolytic genes in *S. cerevisiae*^59^. The codon-optimized sequence, flanked by the constitutive *TPI1* promoter and *CYC1* terminator, was synthesized by GeneArt GmbH (Regensburg, Germany). After subsequent transformation of the pMK-RQ (GeneArt) based vector pUDR350 into *E. coli,* nine different expression cassettes of *xylA* were made, flanked by 60 bp synthetic recombinant sequences (Supplementary Fig. S7). For XylA expression in *E. coli*, a codon-optimized synthetic *xylA* was cloned into pBAD/myc-His-derived plasmid.

Yeast transformation was performed using the lithium acetate protocol^60^. Strain IMX696 was obtained by adding 200 pmol of each of the 15 fragments combined with 500 ng of plasmid pUDE335. After one hour of incubation in synthetic medium with glucose (SMD) the cells were plated on SM plates with xylose as the carbon source (SMX). Correct assembly of all fragments in the *GRE3* locus was confirmed by diagnostic PCR (Dreamtaq, Thermo Scientific) using primers listed in Table S3. Deletion of *PMR1* in *S. cerevisiae* strains IMX696 and CEN.PK113-7D was done by integrating an *amdSYM*-based deletion cassette^61^, which was derived by PCR amplification from pUG-*amdSYM* using primers 8638/8639 as template. After transformation, cells were plated on glucose synthetic medium with acetamide as the nitrogen source (SMD-Ac). Gene deletion was confirmed by diagnostic PCR and the resulting strains were named IMX906 and IMK692, respectively. To reintegrate *PMR1*, the *PMR1* ORF was PCR-amplified from CEN.PK113-7D and transformed into strain IMX906. After overnight incubation in SMD-Ac, cells were plated on SMD plates supplemented with 2.3 g l^−1^ fluoroacetamide (SMD-Fac). Correct integration of *PMR1* in the resulting strain, IMX969, was confirmed by diagnostic PCR.

### Cultivation and media

Shake-flask cultures were grown at 30 °C in an orbital shaker at 200 rpm, using 500-ml flasks containing 100 ml medium. Physiological characterization of aerobic growth was performed in shake flasks containing SMX or SMD with urea as sole nitrogen source to prevent acidification. Prior to filter sterilization, media were adjusted to pH 5.0 with 2 M KOH. For pre-cultures, SM adjusted to pH 6.0 was autoclaved at 12 °C for 20 min after which a 50 w/v % solution of sterile glucose or xylose was added to obtain a final sugar concentration of 20 g l^−1^, together with filter-sterilized vitamin solution^62^. Glucose and xylose solutions were autoclaved separately (20 min at 110 °C). For plates, 2% agar was added to media prior to autoclaving. Frozen stocks (1 ml aliquots in 30% glycerol) were inoculated directly into pre-culture shake flasks. In late exponential phase an aliquot was transferred to a second pre-culture to obtain an initial OD_660_ of 0.1. Flasks or anaerobic bioreactors used for characterization were inoculated from these cultures at an initial OD_660_ of between 0.1 and 0.2. Anaerobic batch cultures were conducted in 2-l bioreactors (Applikon, Delft, The Netherlands) with a working volume of 1 l. Biomass for metal content analysis was grown in 3-l bioreactors (Applikon) with a working volume of 2 l were used. Bioreactor cultures were grown at 30 °C, pH 5.0, and stirred at 800 rpm. To ensure anaerobic conditions, bioreactors were equipped with Viton O-rings and Norprene tubing. During cultivation, nitrogen gas (<10 ppm oxygen) was continuously sparged through the cultures at 0.5 l min^−1^. After autoclaving, synthetic medium used for anaerobic cultivation was supplemented with 0.2 g l^−1^ sterile antifoam C (Sigma-Aldrich), as well as Tween 80 (420 mg l^−1^) and ergosterol (10 mg l^−1^) dissolved in ethanol^63^.

### Analytical methods

Cell dry weight (CDW) measurements were done using pre-weighed nitrocellulose filters (pore size, 0.45 μm; Gelman Laboratory, Ann Arbor, MI) to filter 10 ml of culture. Before weighing the sample, filters were washed with demineralised water and dried in a microwave oven (Bosch, Stuttgart, Germany) for 20 min at 360 W. Growth was monitored by optical density (OD) measurements at a wavelength of 660 nm using a Libra S11 spectrophotometer (Biochrom, Cambridge, United Kingdom). A correlation between OD measurements and CDW was used to estimate CDW in samples for which no direct CDW measurements were taken. This correlation was based on at least six points during the exponential phase.

CO_2_ and O_2_ concentrations in bioreactor exhaust gas were measured using an NGA 2000 analyzer (Rosemount Analytical, Orrville, OH) after the gas was cooled by a condenser (2 °C) and dried with a Permapure MD-110-48P-4 dryer (Permapure, Toms River, NJ). Metabolite levels in culture supernatants obtained by centrifugation were measured via high-performance liquid chromatography (HPLC) analysis on an Agilent 1260 HPLC (Agilent Technologies, Santa Clara, CA) fitted with a Bio-Rad HPX 87 H column (Bio-Rad, Hercules, CA). The column was eluted at 60 °C with 0.5 g l^−1^ H_2_SO_4_ at a flow rate of 0.6 ml min^−1^. Detection was by means of an Agilent refractive-index detector and an Agilent 1260 VWD detector. Correction for ethanol evaporation were done for all bioreactor experiments as described previously^64^.

Viability of strain IMX696 during anaerobic cultivation was assessed by plating culture samples. The number of cells per ml was measured using a Z2 Coulter Counter (Beckman Coulter, Woerden, The Netherlands) after which dilutions were plated in duplicate on SMX and SMG agar plates and incubated at 30 °C. To limit exposure to oxygen, cells that were used to determine anaerobic viability measurements were sampled directly into a container flushed with argon and immediately transferred into an anaerobic chamber (5% H_2_, 6% CO_2_, and 89% N_2_, Sheldon MFG Inc., Cornelius, OR) for plating and incubation. Colony-forming units (CFU) were counted after incubation at 30 °C for 4 days (aerobic growth) or 8 days (anaerobic growth).

### DNA sequence analysis

Genomic DNA of strains IMX696, IMS0488 and IMS0489 was isolated using the QIAGEN Blood & Cell Culture DNA Kit with 100/G Genomics-tips (QIAGEN, Valencia, CA) according to the manufacturer’s protocol. From these DNA samples, 350-bp insert libraries were constructed using the Nextera XT DNA kit (Illumina, San Diego, CA). Paired-end sequencing (100-bp reads) of genomic or plasmid DNA was performed with an Illumina HiSeq 2500 sequencer (Baseclear BV, Leiden, The Netherlands). Data were mapped to the CEN.PK113-7D genome or to in silico-generated plasmid sequences using the Burrows-Wheeler alignment tool^65^ and processed with Pilon^66^. Identified single-nucleotide differences were inspected with the Integrated Genomics Viewer^67^ (IGV). The chromosomal copy number variance (CNV) was estimated using the Poisson mixture model based algorithm Magnolya^68^. The copy number of *xylA* was estimated by comparing the read depth to the average read depth of all chromosomes. Raw sequence data of strains IMX696, IMS0488 and IMS0489 are deposited at the NCBI Sequence Read archive (www.ncbi.nlm.nih.gov/sra) under BioProject ID PRJNA349142.

### Purification of xylose isomerase

Cell pellets were resuspended in 10 mM MOPS, pH 7.0, containing protease inhibitors (cOmplete ULTRA tablets, Roche) and disrupted using a high pressure homogenizer (Constant Systems Ltd, Low March, United Kingdom). Samples were passed through the apparatus twice at 39 kpsi and cell debris was removed by centrifugation at 35,000 × g for 45 min at 4 °C. A single-step purification procedure based on anion-exchange chromatography was applied to minimize the loss of protein-bound metals. Cell-free extracts were loaded on a strong anion-exchange column (Resource Q, GE Healthcare, Chicago, IL) equilibrated with 10 mM MOPS, pH 7.0. A gradient elution was applied using 10 mM MOPS, pH 7.0, containing 0–200 mM KCl. XylA eluted at approximately 40 mM KCl. Protein concentrations were determined using the theoretical extinction coefficient at 280 nm (ε280, XI = 73,800 M^−1^ cm^−1^) calculated by the ProtParam tool (http://web.expasy.org/protparam/).

### Metal content analysis

Metal concentrations were analysed with an inductively coupled plasma mass spectrometer (ICP-MS, Varian 820). All measurements were performed 5 times for each sample and yttrium was used as an internal standard. Purified protein samples were lyophilized and analysed for contents of magnesium, calcium, iron and manganese. Prior to measurement, samples were dissolved in 1% nitric acid solution. All analyses were performed on protein samples isolated from two replicate cultures. For intracellular metal analysis, cells were prepared with a protocol adopted from Eide *et al*.^69^. The harvested cells were washed three times each with 1 μM EDTA solution and subsequently with deionized water (Milli-Q) and suspended in 1 ml 30% (w/v) nitric acid and incubated at 6 °C for 4 h. Cell lysates were centrifuged at 16,000 × g and supernatants were collected. Pellets were washed with 1 ml deionized water and the supernatants were collected as before. The 2 ml of final sample solution containing approximately 15% (w/v) nitric acid were then subjected to the measurements. The metal content was determined with samples from two separate bioreactor batch cultures.

### Preparation of cell extracts

Cell extracts were prepared following a previously published procedure with minor modifications^16^. To limit loss of metals during preparation, no EDTA was added prior to sonication. Cells were washed and suspended in 10 mM MOPS buffer pH 7.0 to avoid precipitation of MnCl_2_ and 10 mM DTT was added. After sonication (4 bursts of 30 s with 30 s intervals at 0 °C, amplitude 8 μm) using a Soniprep 150 sonicator (Beun de Ronde BV, Abcoude, The Netherlands), cell debris was removed by centrifugation (4 °C, 20 min at 48,000 g) and the clear supernatant was used for XylA assays.

### Enzyme activity assays

Activity of XylA was measured with a coupled enzyme assay using d-sorbitol dehydrogenase^70^. d-sorbitol dehydrogenase (SDH) was obtained from Roche Diagnostics GmbH (Mannheim, Germany). Reactions were performed at 30 °C and pH 7.0 (20 mM MOPS buffer). The decrease in absorbance at 340 nm was monitored in either a spectrophotometer (Jasco, Easton, MD) or a Synergy Mx microtiter plate reader (BioTek Instruments, Winooski, VT). Reaction mixtures included 5, 200 or 500 mM xylose, 250 μM NADH and  U ml^−1^ of SDH. Addition of 0.03 to 1 μM (depending on the substrate concentration and the metal added) XI or cell free extract into the mixture initiated the reaction. For measuring XylA activity in the presence of different metal cofactors, samples of apo*-*XylA were prepared by overnight incubation of the purified enzyme with 10 mM EDTA. Subsequently, EDTA was removed by buffer exchange to 20 mM MOPS, pH 7.0 and 1 mM of divalent metal solutions (MgCl_2_, MnCl_2_ or CaCl_2_) were added in the reaction. For kinetic analyses, d-xylose was added at concentrations ranging from 0.5 mM to 1.50 M.

## Additional Information

**How to cite this article**: Verhoeven, M. D. *et al*. Mutations in *PMR1* stimulate xylose isomerase activity and anaerobic growth on xylose of engineered *Saccharomyces cerevisiae* by influencing manganese homeostasis. *Sci. Rep.* **7**, 46155; doi: 10.1038/srep46155 (2017).

**Publisher's note:** Springer Nature remains neutral with regard to jurisdictional claims in published maps and institutional affiliations.

## Supplementary Material

Supplementary Tables and Figures

## Figures and Tables

**Table 1:** *Saccharomyces cerevisiae* strains used in this study.

Strain	Relevant genotype/description	Reference
CEN.PK 113-7D	*MATa MAL2-8c SUC2*	55
IMX581	*MATa ura3-52 MAL2-8c SUC2 can1*Δ::*cas9-*natNT2	33
IMX696	*MATa ura3-52 MAL2-8c SUC2 CAN1::cas9-*natNT2 *gre3::* [pTDH3_RPE1- pPGK1_TKL1- pTEF1_TAL1- pPGI1_NQM - pTPI1_RKI1- pPYK1_TKL2- (pTPI1_xylA_tCYC1)*36 pTEF1_XKS1] pUDE335	This study
IMS0488	Single-cell line isolated after adaptation of IMX696 to anaerobic growth on xylose (reactor 1)	This study
IMS0489	Single-cell line isolated after adaptation of IMX696 to anaerobic growth on xylose (reactor 2)	This study
IMX906	*MATa ura3-52 MAL2-8c SUC2 CAN1::cas9-*natNT2 gre3:: [pTDH3_RPE1- pPGK1_TKL1- pTEF1_TAL1- pPGI1_ NQM1-pTPI1_RKI1- pPYK1_TKL2- (pTPI1_xylA_tCYC1)*36 *pTEF1_XKS1] pmr1*Δ::*amdSYM* pUDE335	This study
IMX979	IMX906 with *PMR1* reintegrated at *PMR1* locus	This study
IMK692	*MATa MAL2-8c SUC2 pmr1*Δ::*amdSYM*	This study

Native gene terminator sequences were used for expression of *RPE1, TKL1, TAL1, NQM1, RKI1, TKL2* and *XKS1*.

**Table 2:** Single-nucleotide mutations in engineered *S. cerevisiae* strains adapted to anaerobic growth on xylose.

Strain	Gene	Nucleotide change	Amino acid change	Change in codon
IMS0488	*PMR1*	G746T	G249V	gGt/gTt
IMS0489	*PMR1*	G1161A	W387*	tgG/tgA

Strains IMS0488 and IMS0489 were isolated from independent anaerobic batch cultures of strain IMX696 (*xylA*, PPP↑, *XKS1*↑). The genome sequence of IMX696 was used as a reference. *Introduction of stop codon.

**Table 3:** Impact of the deletion of *PMR1* on intracellular metal ion concentrations.

*S. cerevisiae* strain (relevant genotype)	Carbon source	μmol (g dry biomass)^−1^				
		Mg^2+^	Ca^2+^	Fe^2+^	Mn^2+^	Total
IMX696 (*xylA, PPP*↑, *XKS1*↑)	glucose	60 ± 1	8.8 ± 1.6	0.56 ± 0.02	0.069 ± 0.001	69 ± 2
IMX906 (*xylA, PPP*↑, *XKS1*↑, *pmr1*Δ)	glucose	64 ± 1	12 ± 2	0.51 ± 0.02	1.2 ± 0.1	77 ± 2
IMX906 (*xylA*, PPP↑, *XKS1*↑, *pmr1*Δ)	xylose	50 ± 1	4.0 ± 0.7	0.36 ± 0.01	1.4 ± 0.1	56 ± 1
CEN.PK113-7D (reference strain)	glucose	61 ± 1	6.8 ± 1.3	0.44 ± 0.01	0.046 ± 0.001	68 ± 2
IMK692 (*pmr1*Δ)	glucose	72 ± 1	6.7 ± 1.2	0.46 ± 0.02	0.86 ± 0.06	80 ± 2

*S. cerevisiae* strains were grown in anaerobic bioreactors on xylose or glucose (20 g l^−1^). Data represent average and mean deviation calculated from analyses on independent duplicate cultures.

**Table 4:** Impact of *PMR1* deletion on metal content and activity of XylA.

*S. cerevisiae* strain (relevant genotype)	Carbon source	mol metal/mol XylA monomer				Sp. activity (U/mg XylA protein)	
		Mg^2+^	Ca^2+^	Fe^2+^	Mn^2+^	no metal added	Mg^2+^ added (1 mM)
IMX906 (*xylA*, PPP↑, *XKS1*↑, *pmr1*Δ)	xylose	0.18 ± 0.01	0.54 ± 0.11	0.06 ± 0.01	0.38 ± 0.04	2.39 ± 0.61	2.68 ± 0.5
IMX906 (*xylA*, PPP↑, *XKS1*↑, *pmr1*Δ)	glucose	0.20 ± 0.06	0.66 ± 0.07	0.064 ± 0.02	0.30 ± 0.03	1.35 ± 0.14	2.10 ± 0.1
IMX696 (*xylA*, PPP↑, *XKS1*↑)	glucose	0.22 ± 0.06	0.93 ± 0.41	0.086 ± 0.04	0.017 ± 0.004	0.60 ± 0.24	1.68 ± 0.1

XylA protein was isolated from *S. cerevisiae* cultures grown on xylose or glucose (20 g l^−1^) in anaerobic bioreactors. Data represent average and mean deviation of analyses on XylA isolated from independent duplicate cultures.

**Table 5:** Kinetic parameters of XylA measured after reconstituting apo-XylA with different divalent metal ions.

Metal	Kinetic parameters		
	*k*_cat_ (s^−1^)	*K*_M_ (mM)	*k*_cat_/*K*_M_ (s^−1^ · M^−1^)
Mg^2+^	2.8 ± 0.2	5.5 ± 0.4	500
Mn^2+^	7.8 ± 0.1	3.9 ± 0.2	2000
Ca^2+^	0.6 ± 0.06	420 ± 90	1.3

*k*_cat_ and *K*_M_ values represent average and mean deviation of independent duplicate experiments, calculated for each metal.
