# Supplementary material for: Mutations in PMR1 stimulate xylose isomerase activity and anaerobic growth on xylose of engineered Saccharomyces cerevisiae by influencing manganese homeostasis
Source: Sci Rep. 2017 Apr 12;7:46155. doi: 10.1038/srep46155 (PMC5388867; doi:10.1038/srep46155)
Supplement: Supplementary Tables and Figures [file srep46155-s1.pdf]

Supplementary information

Mutations in *PMR1* stimulate xylose isomerase activity and anaerobic growth on xylose of engineered *Saccharomyces cerevisiae* by influencing manganese homeostasis

Maarten D. Verhoeven[1,+], Misun Lee [2,+], Lycka Kamoen [1], Marcel van den Broek [1], Dick B. Janssen[2], Jean-Marc G. Daran[1], Antonius J.A. van Maris[1,†] & Jack T. Pronk[1,*]

[1]Department of Biotechnology, Delft University of Technology, Van der Maasweg 9, 2629 HZ Delft, The Netherlands

[2]Department of Biochemistry, Groningen Biomolecular Sciences and Biotechnology Institute (GBB), University of Groningen, Nijenborgh 4, 9747 AG Groningen, The Netherlands

[+]These authors contributed equally to this work

[†]Current address: Division of Industrial Biotechnology, School of Biotechnology, KTH Royal Institute of Technology, AlbaNova University Center, SE 106 91, Stockholm, Sweden.

*Corresponding author: Jack Pronk, Delft University of Technology, Van der Maasweg 9, 2629 HZ Delft, The Netherlands. Email j.t.pronk@tudelft.nl, Tel: +31152783214, Fax: +31152702355

Table S1. Activity of XylA in the presence of different metal mixtures.

	XylA activities (U·(mg protein)$^{-1}$)			
Xylose (mM)	Mg^{2+}	Mn^{2+}	Mix 1<br>Mg:Ca:Mn<br>0.85:0.15:0.001	Mix 2<br>Mg:Ca:Mn<br>0.84:0.14:0.02
5	1.73 ± 0.19	4.03 ± 0.44	1.87 ± 0.42	3.53 ± 0.73
200	3.13 ± 0.10	7.05 ± 0.67	3.35 ± 0.06	6.02 ± 0.45

XylA was expressed in *E. coli*, purified and EDTA-treated. Enzyme activities were measured in the presence different metal (mixtures), total concentration 1 mM. The molar fractions in metal mixes 1 and 2 represent the intracellular metal composition of IMX696 and IMX906, respectively (see Table 3 and main text). Reactions were performed at 30 ˚C and pH 7.0 with saturating concentration (200 mM) and near K_M (5 mM) of xylose. The values represent average and mean deviation of measurements with XylA isolated from independent duplicate *E. coli* cultures.

Table S2. Plasmids used in this study.

Plasmid	Characteristics	Origin
**pMEL10**	2 µm ori, *KlURA3*, p*SNR52*-gRNA.CAN1.Y-t*SUP4*	Mans *et al.* (2015)[33]
**pJET1.2Blunt**	Multi-purpose cloning vector	ThermoFisher
**pUD344**	pJET1.2Blunt + TagA_p*PGI1_NQM1*_TagB	This study
**pUD345**	pJET1.2Blunt + TagB_p*TPI1_RKI1*_Tagc	This study
**pUD346**	pJET1.2Blunt + TagC_p*PYK1_TKL2*_TagF	This study
**pUD347**	pJET1.2Blunt + TagG_p*TDH3_RPE1*_TagH	This study
**pUD348**	pJET1.2Blunt + TagH_p*PGK1_TKL1*_TagI	This study
**pUD349**	pJET1.2Blunt + TagI_p*TEF1_TAL1*_TagA	This study
**pUD350**	pMK-RQ_p*TPI1_xylA*_t*cyc*	This study
**pUD353**	pJET_Blunt_p*TEF1_XKS1*_t*XKS1*	This study
**pUDE335**	2 µm ori, *KlURA3*, p*SNR52*-gRNA.*GRE3*.Y-t*SUP4*	This study
**pUG-AmdSYM**	Template of AmdSYM cassette for *PMR1* deletion	Solis-Escalante *et al.* (2013)[63]

Native gene terminator sequences were used for expression of *RPE1, TKL1, TAL1, NQM1, RKI1, TKL2* and *XKS1*.

Table S3. Oligonucleotide primers used in this study.

Primer nr.:	Purpose:	Template:	Sequence 5' -> 3':
**Construction of cassettes containing constitutively expressed-pentose-phosphate-pathway and *XKS1* genes:**			
5924	*PGI1* promoter fragment	CEN.PK113-7D	ACTATATGTGAAGGCATGGCTATGGCACGGCAGACATTCCGCCAGATCATCAATAGGCACCAGGGCACTACTTCTACACATCAACG
5925	*PGI1* promoter fragment	CEN.PK113-7D	TTTTAGGCTGGTATCTTGATTCTAAATCG
5926	*NQM1* ORF fragment	CEN.PK113-7D	TCGATTTAGAATCAAGATACCAGCCTAAAAATGTCAGAACCTTCAGAGAAAAAAC
5927	*NQM1* ORF fragment	CEN.PK113-7D	GTTGAACATTCTTAGGCTGGTCGAATCATTTAGACACGGGCATCGTCCTCTCGAAAGGTGGCCCAAGAGGATATTAAGTACTAATGTGG
3847	fusion-PCR of pPGI1 and *NQM1*	pPGI1 + *NQM1* fragment	ACTATATGTGAAGGCATGGCTATGG
3276	fusion-PCR of pPGI1 and *NQM1*	pPGI1 + *NQM1* fragment	GTTGAACATTCTTAGGCTGGTCGAATC
5928	*TPI1* promoter fragment	CEN.PK113-7D	CACCTTTCGAGAGGACGATGCCCGTGTCTAAATGATTCGACCAGCCTAAGAATGTTCAACGCGGCCGTGTTTAAAGATTAC
5929	*TPI1* promoter fragment	CEN.PK113-7D	CCGCGGAGTTTATGTATG
5930	*RKI1* ORF fragment	CEN.PK113-7D	CTTAAATCTATAACTACAAAAAACACATACATAAACTCCGCGGATGGCTGCCGGTGTCCC
5931	*RKI1* ORF fragment	CEN.PK113-7D	CTAGCGTGTCCTCGCATAGTTCTTAGATTGTCGCTACGGCATATACGATCCGTGAGACGTATCATAGGTGAGAAAGAGATGGAGAATGTAGTACTGC
4672	fusion-PCR of pTPI1 and *RKI1*	pTPI1+ *RKI1* fragment	CACCTTTCGAGAGGACGATG
3277	fusion-PCR of pTPI1 and *RKI1*	pTPI1+ *RKI1* fragment	CTAGCGTGTCCTCGCATAGTTCTTAGATTG
5932	*PYK1* promoter fragment	CEN.PK113-7D	ACGTCTCACGGATCGTATATGCCGTAGCGACAATCTAAGAACTATGCGAGGACACGCTAGGGTAGCGCCCTGGTCAAACTTCAGAAC
5933	*PYK1* promoter fragment	CEN.PK113-7D	TGTGATGATGTTTTATTTGTTTTGATTGGTGTC
5934	*TKL2* ORF fragment	CEN.PK113-7D	CACCAATCAAAACAAATAAAACATCATCACAATGGCACAGTTCTCCGACATTGATAAACTTGC
5935	*TKL2* ORF fragment	CEN.PK113-7D	TGCCGAACTTTCCCTGTATGAAGCGATCTGACCAATCCTTTGCCGTAGTTTCAACGTATGGCAGCCCATACACTCAAAGC
3283	fusion-PCR of pPYK1 and *TKL2*	pPYK1 + *TKL2* fragment	ACGTCTCACGGATCGTATATGC
3288	fusion-PCR of pPYK1 and *TKL2*	pPYK1 + *TKL2* fragment	TGCCGAACTTTCCCTGTATGAAGC
5912	*TDH3* promoter fragment	CEN.PK113-7D	GCCAGAGGTATAGACATAGCCAGACCTACCTAATTGGTGCATCAGGTGGTCATGGCCCTTCCGGGAGTTTATCATTATCAATACTCG
5913	*TDH3* promoter fragment	CEN.PK113-7D	CCGTCGAAACTAAGTTCTTGG
5914	*RPE1* ORF fragment	CEN.PK113-7D	TTAGTTTTAAAACACCAAGAACTTAGTTTCGACGGATGGTCAAACCAATTATAGCTCCCAGTATCC
5915	*RPE1* ORF fragment	CEN.PK113-7D	GTCACGGGTTCTCAGCAATTCGAGCTATTACCGATGATGGCTGAGGCGTTAGAGTAATCTCTTCTCCGGCCTCCATCACCAC
4870	fusion-PCR of pTDH3and *RPE1*	pTDH3 + *RPE1* fragment	GCCAGAGGTATAGACATAGCC
3290	fusion-PCR of pTDH3and *RPE1*	pTDH3 + *RPE1* fragment	GTCACGGGTTCTCAGCAATTCG
5916	*PGK1* promoter fragment	CEN.PK113-7D	AGATTACTCTAACGCCTCAGCCATCATCGGTAATAGC

			TCGAATTGCTGAGAACCCGTGACTGCCCTTATCTTGT GCAGTTAGAC
5917	*PGK1* promoter fragment	CEN.PK113-7D	TGTTTTATATTTGTTGTAAAAAGTAGATAATTACTTCC
5918	*TKL1* ORF fragment	CEN.PK113-7D	GGAAGTAATTATCTACTTTTTACAACAAATATAAAAC AATGACTCAATTCACTGACATTGATAAGC
5919	*TKL1* ORF fragment	CEN.PK113-7D	GCCTACGGTTCCCGAAGTATGCTGCTGATGTCTGGC TATACCTATCCGTCTACGTGAATAATGAATGCGACCG ATATTTTTGG
3291	fusion-PCR of pPGK1 and *TKL1*	pPGK1 + *TKL1* fragment	CTCTAACGCCTCAGCCATCATCG
4068	fusion-PCR of pPGK1 and *TKL1*	pPGK1 + *TKL1* fragment	GCCTACGGTTCCCGAAGTATGC
5920	*TEF1* promoter fragment	pYM-N18	TATTCACGTAGACGGATAGGTATAGCCAGACATCAG CAGCATACTTCGGGAACCGTAGGCAGCTCATAGCTT CAAAATGTTTCTACTCC
5921	*TEF1* promoter fragment	pYM-N18	AAAACTTAGATTAGATTGCTATGCTTTCTTTCTAATG AGC
5922	*TAL1* ORF fragment	CEN.PK113-7D	GCTCATTAGAAAGAAAGCATAGCAATCTAATCTAAG TTTTATGTCTGAACCAGCTCAAAAGAAACAAAAGG
5923	*TAL1* ORF fragment	CEN.PK113-7D	GTGCCTATTGATGATCTGGCGGAATGTCTGCCGTGC CATAGCCATGCCTTCACATATAGTCATTGTGATCCTC CTATGTTGTAGTATAGTGC
3274	fusion-PCR of pTEF1 and *TAL1*	pTEF1+ *TAL1* fragment	TATTCACGTAGACGGATAGGTATAGC
3275	fusion-PCR of pTEF1 and *TAL1*	pTEF1+ *TAL1* fragment	GTGCCTATTGATGATCTGGCGGAATG
6278	*XKS1* ORF fragment	CEN.PK113-7D	GCAATGACAAATTCAAAAGAAGACGCCGACATAGA GGAGAAGCATATGTACAATGAGCCGGTCCAGTGCTT CCACATC
6279	*XKS1* ORF fragment	CEN.PK113-7D	GCTCATTAGAAAGAAAGCATAGCAATCTAATCTAAG TTTTATGTTGTGTTCAGTAATTCAGAGACAG

Primers used for amplification of integration fragments:

Primer nr.:	Purpose:	Template:	Sequence:
7133	fl_*RPE1*_H fragment fw	pUD347	TATAATATTTCATTATCGGAACTCTAGATTCTATACTTGTTTCCCA ATTGTTGCTGGTAGGGCCCTTCCGGGAGTTTATC
3290	fl_*RPE1*_H fragment rv	pUD347	GTCACGGGTTCTCAGCAATTCG
3291	H_*TKL1*_I fragment fw	pUD348	CTCTAACGCCTCAGCCATCATCG
4068	H_*TKL1*_I fragment rv	pUD348	GCCTACGGTTCCCGAAGTATGC
3274	I_*TAL1*_A fragment	pUD349	TATTCACGTAGACGGATAGGTATAGC
3275	I_*TAL1*_A fragment	pUD349	GTGCCTATTGATGATCTGGCGGAATG
3847	A_ *NQM1* _B	pUD344	ACTATATGTGAAGGCATGGCTATGG
3276	A_ *NQM1* _B	pUD344	GTTGAACATTCTTAGGCTGGTCGAATC
4672	B_*RKI1*_C fragment	pUD345	CACCTTTCGAGAGGACGATG
3277	B_*RKI1*_C fragment	pUD345	CTAGCGTGTCCTCGCATAGTTCTTAGATTG
3283	C_*TKL2*_F fragment	pUD346	ACGTCTCACGGATCGTATATGC
3288	C_*TKL2*_F fragment	pUD346	TGCCGAACTTTCCCTGTATGAAGC
7138	F_*xylA*_P fragment	pUD350	CTGATAGTGCTGTAAGTCGCCTCCATCTTAGCAGAGCTGTCCCT GAATGCGTACTCGTGAGCGATACCCTGCGATCTTC
7136	F_*xylA*_P fragment	pUD350	CATACGTTGAAACTACGGCAAAGGATTGGTCAGATCGCTTCATA CAGGGAAAGTTCGGCACGCGCAGATTAGCGAAGC
7139	P_*xylA*_Q fragment	pUD350	GAGCTGAATGTATATGCTGCGGGATCATTGCACAGCTCTGAGA GCCCTGCAACGCGATATGCGATACCCTGCGATCTTC
7137	P_*xylA*_Q fragment	pUD350	TCACGAGTACGCATTCAGGGACAGCTCTGCTAAGATGGAGGCG ACTTACAGCACTATCAGCGCGCAGATTAGCGAAGC

7142	Q_*xylA*_E fragment	pUD350	AGCGATCTGCGAGACCGTATAGCCATGACGAGGTCGCAATCTTG CGGACAGTGTAGCTCAGCGATACCCTGCGATCTTC
7140	Q_*xylA*_E fragment	pUD350	ATATCGCGTTGCAGGGCTCTCAGAGCTGTGCAATGATCCCGCAG CATATACATTCAGCTCCGCGCAGATTAGCGAAGC
7141	E_*xylA*_G fragment	pUD350	TGAGCTACACTGTCCGCAAGATTGCGACCTCGTCATGGCTATAC GGTCTCGCAGATCGCTCGCGCAGATTAGCGAAGC
6285	E_*xylA*_G fragment	pUD350	AAGGGCCATGACCACCTGATGCACCAATTAGGTAGGTCTGGCTA TGTCTATACCTCTGGCGCGATACCCTGCGATCTTC
6273	G_*xylA*_D fragment	pUD350	GCCAGAGGTATAGACATAGCCAGACCTACCTAATTGGTGCATCA GGTGGTCATGGCCCTTCGCGCAGATTAGCGAAGC
6284	G_*xylA*_D fragment	pUD350	AATCACTCTCCATACAGGGTTTCATACATTTCTCCACGGGACCCA CAGTCGTAGATGCGTGCGATACCCTGCGATCTTC
6283	D_*xylA*_M fragment	pUD350	ACGCATCTACGACTGTGGGTCCCGTGGAGAAATGTATGAAACCC TGTATGGAGAGTGATTGCGATACCCTGCGATCTTC
6275	D_*xylA*_M fragment	pUD350	ACGAGAGATGAAGGCTCACCGATGGACTTAGTATGATGCCATG CTGGAAGCTCCGGTCATCGCGCAGATTAGCGAAGC
6287	M_*xylA*_N fragment	pUD350	ATGACCGGAGCTTCCAGCATGGCATCATACTAAGTCCATCGGTG AGCCTTCATCTCTCGTGCGATACCCTGCGATCTTC
6276	M_*xylA*_N fragment	pUD350	TTCTAGGCTTTGATGCAAGGTCCACATATCTTCGTTAGGACTCAA TCGTGGCTGCTGATCCGCGCAGATTAGCGAAGC
6288	N_*xylA*_O fragment	pUD350	GATCAGCAGCCACGATTGAGTCCTAACGAAGATATGTGGACCTT GCATCAAAGCCTAGAAGCGATACCCTGCGATCTTC
6277	N_*xylA*_O fragment	pUD350	ATACTCCCTGCACAGATGAGTCAAGCTATTGAACACCGAGAACG CGCTGAACGATCATTCCGCGCAGATTAGCGAAGC
6289	O_*xylA*_L fragment	pUD350	GAATGATCGTTCAGCGCGTTCTCGGTGTTCAATAGCTTGACTCAT CTGTGCAGGGAGTATGCGATACCCTGCGATCTTC
6627	O_*xylA*_L fragment	pUD350	GCCGTAGCTTCCGCAAGTATGCCGTAGTTGAAGAGCATTTGCCG TCGGTTCAGGTCATATCGCGCAGATTAGCGAAGC
7135	L_*XKS1*_fl fragment	pUD353	ATATGACCTGAACCGACGGCAAATGCTCTTCAACTACGGCATAC TTGCGGAAGCTACGGCCATAGCTTCAAAATGTTTCTACTCC
7134	L_*XKS1*_fl fragment	pUD353	TGTGGCACCGCAATCATTACTATGGCTAGTGCTATCATTGCTGTT TGACGCACTGATGGGGTCCAGTGCTTCCACATCAATTTG
8638	*PMR1* KO cassette	pUG-AmdSYM	CAAGACGAAGCAAGGCCAGCACAGACGTAAGCTTAAGTGTAAG TAAAAGATAAGATAATTCAGCTGAAGCTTCGTACGC
8639	*PMR1* KO cassette	pUG-AmdSYM	ACATATGTTCCCATTAATTAGTACATTTACCTCAATAGGGTTGGG CGTCCTCATGAAAGAGCATAGGCCACTAGTGGATCTG
8640	*PMR1* reintgration	CEN,PK113-7D	CCATGGCTACTGCTATTTCG
8641	*PMR1* reintgration	CEN,PK113-7D	AGGGCGTTGATAGGATG

Primers used for construction of plasmid containing the gRNA cutting in *GRE3:*

Primer nr.:	Purpose:	Template:	
5792	pUDE335 backbone	pMEL10	GTTTTAGAGCTAGAAATAGCAAGTTAAAATAAG
5980	pUDE335 backbone	pMEL10	CGACCGAGTTGCTCTTG
5978	pUDE335 gRNA	pMEL10	ATTTTAACTTGCTATTTCTAGCTCTAAAACTTTATGACGTATAC GTTTACGATCATTTATCTTTCACTGCGG
5979	pUDE335 gRNA	pMEL10	TATTGACGCCGGGCAAGAGC
2528	restriction analysis of gRNA	pUDE335	TCTTTCCTGCGTTATCCC
960	restriction analysis of gRNA	pUDE335	GTGGATGATGTGGTCTCTAC

Primers used for verifying integration of fragments:

Primer nr.:	Purpose:	Sequence:
6640	checking PPP integration	CTAGATGTGGTCAGCCATTC

976	checking PPP integration	CACCAGTGTCGGCAACAACG
6717	checking PPP integration	CTCATTAGAAAGAAAGCATAGCAATC
5603	checking PPP integration	CGCAAGTTTATCAATGTCGG
4656	checking PPP integration	CCTTCCCATATGATGCTAGG
7056	checking *xylA* integration	AGAGGTGGTGGTTTCGTTAC
6632	checking *xylA* integration	AGCGTCGTAGTAGTGGAAAGC
7370	checking *xylA* integration	TGCTGTAAGTCGCCTCCATC
3293	checking *xylA* integration	GAGCTGAATGTATATGCTGCGGGATC
7369	checking *xylA* integration	AGCGATCTGCGAGACCGTATAG
4692	checking *xylA* integration	AAGGGCCATGACCACCTG
5231	checking *xylA* integration	AATCACTCTCCATACAGGG
3354	checking *xylA* integration	ACGCATCTACGACTGTGGGTC
4184	checking *xylA* integration	ATGACCGGAGCTTCCAGCATG
3843	checking *xylA* integration	GATCAGCAGCCACGATTG
3837	checking *xylA* integration	GAATGATCGTTCAGCGCG
6921	checking *xylA* integration	AGAGGTGGTGGTTTCGTTAC
3286	checking *xylA* integration	GCCGTAGCTTCCGCAAGTATG
8640	checking *PMR1* KO/integration	CCATGGCTACTGCTATTTCG
8641	checking *PMR1* KO/integration	AGGGCGTTGATAGGATG
9	checking *PMR1* KO/integration	CGCACGTCAAGACTGTCAAG
10	checking *PMR1* KO/integration	TCGTATGTGAATGCTGGTCG
8792	checking *PMR1* KO/integration	GTTGGACTGTCTCTGTTAGG
8793	checking *PMR1* KO/integration	CTTCGTCCACGGATAAAG

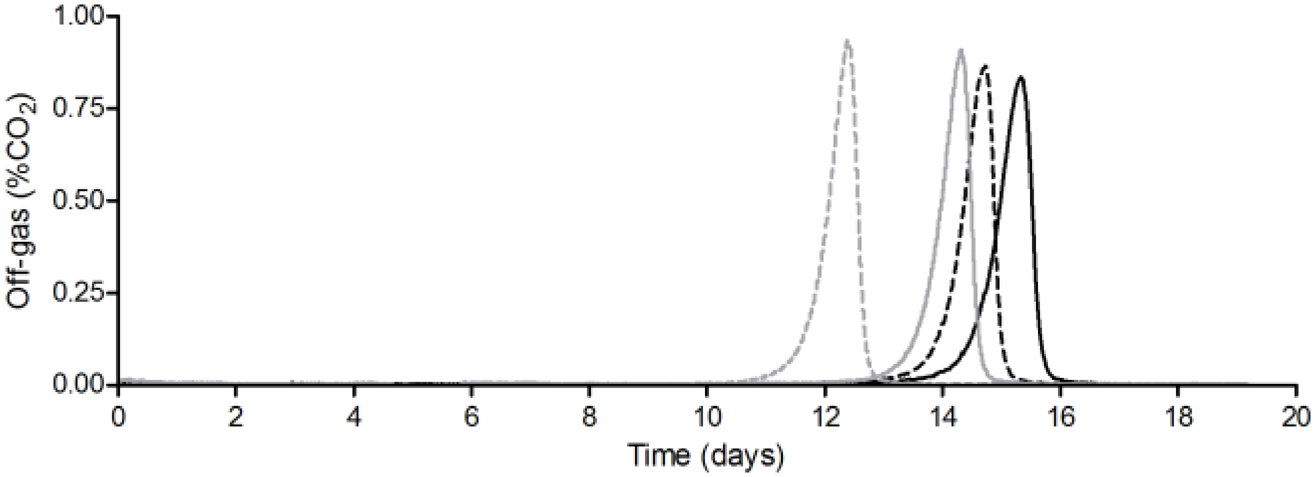

Figure S1│ Off-gas CO_2 profiles of anaerobic bioreactor cultures on synthetic medium with xylose (20 g l^{-1}). Black and grey lines indicate results from independent duplicate cultures of strain *S. cerevisiae* IMX696 (*xylA*, PPP↑, *XKS1*↑) and strain IMX979 (*xylA*, PPP↑, *XKS1*↑, *PMR1*), respectively.

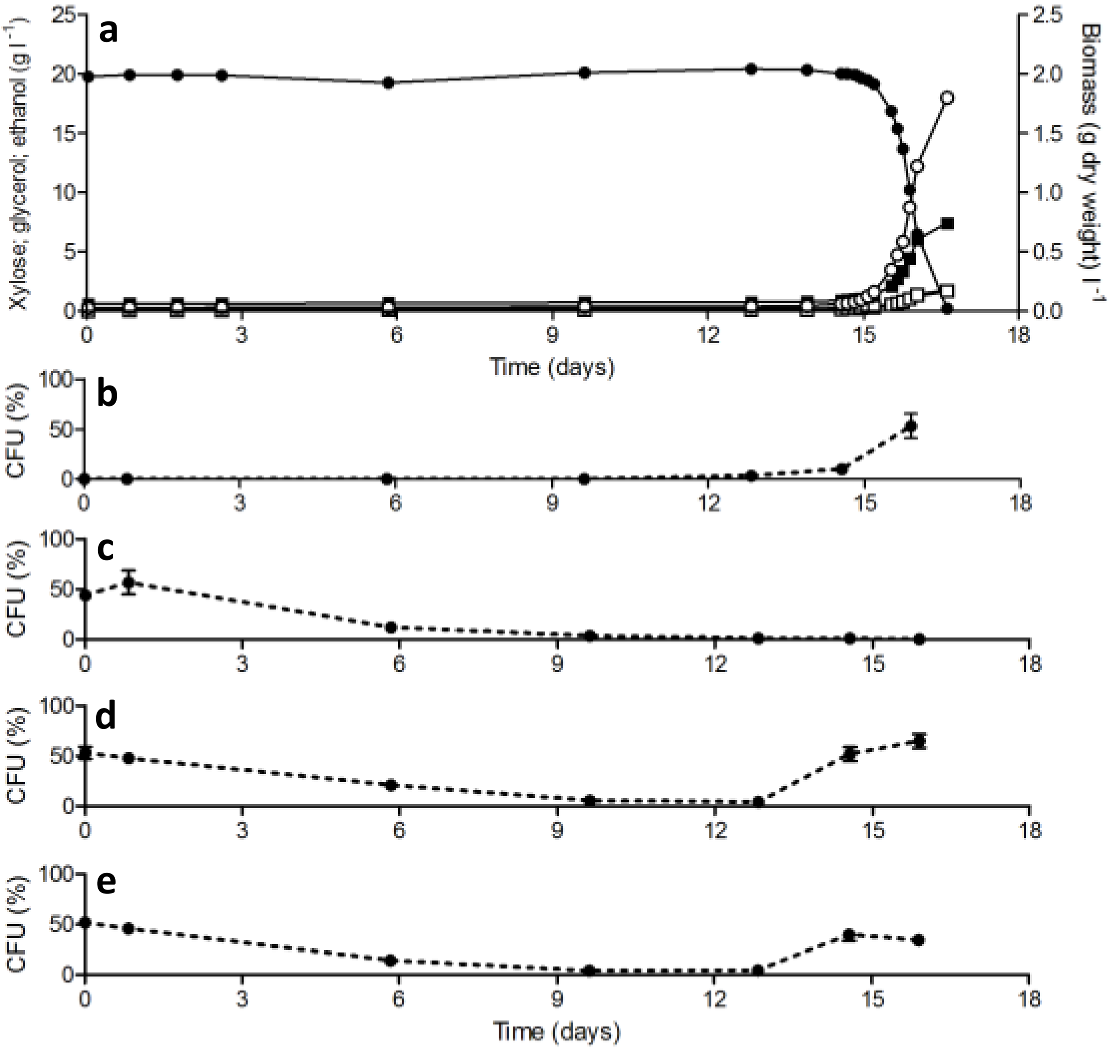

Figure S2 │ Anaerobic growth of *S. cerevisiae* IMX696 (*xylA*, PPP↑, *XKS1*↑) on xylose, independent replicate of experiment shown in Fig. 1. (**a**) Growth, xylose consumption and product formation after inoculation of aerobically pregrown cells in anaerobic bioreactors containing synthetic medium with xylose (20 g l^{-1}). Symbols: ●, xylose, ■, ethanol, ○, biomass, □, glycerol. (**b**) Colony-forming units (CFU) on anaerobically incubated xylose medium reflect adaptation to growth on xylose in the absence of oxygen. (**c**) CFU on aerobically incubated xylose medium reflect trade-off between aerobic and anaerobic growth on xylose. (**d**) and (**e**) CFU on anaerobically and aerobically incubated glucose medium, respectively, showing that oxygen sensitivity of cells adapted to anaerobic growth on xylose is not carbon-source dependent. Data shown in this figure are from one of two independent replicates.

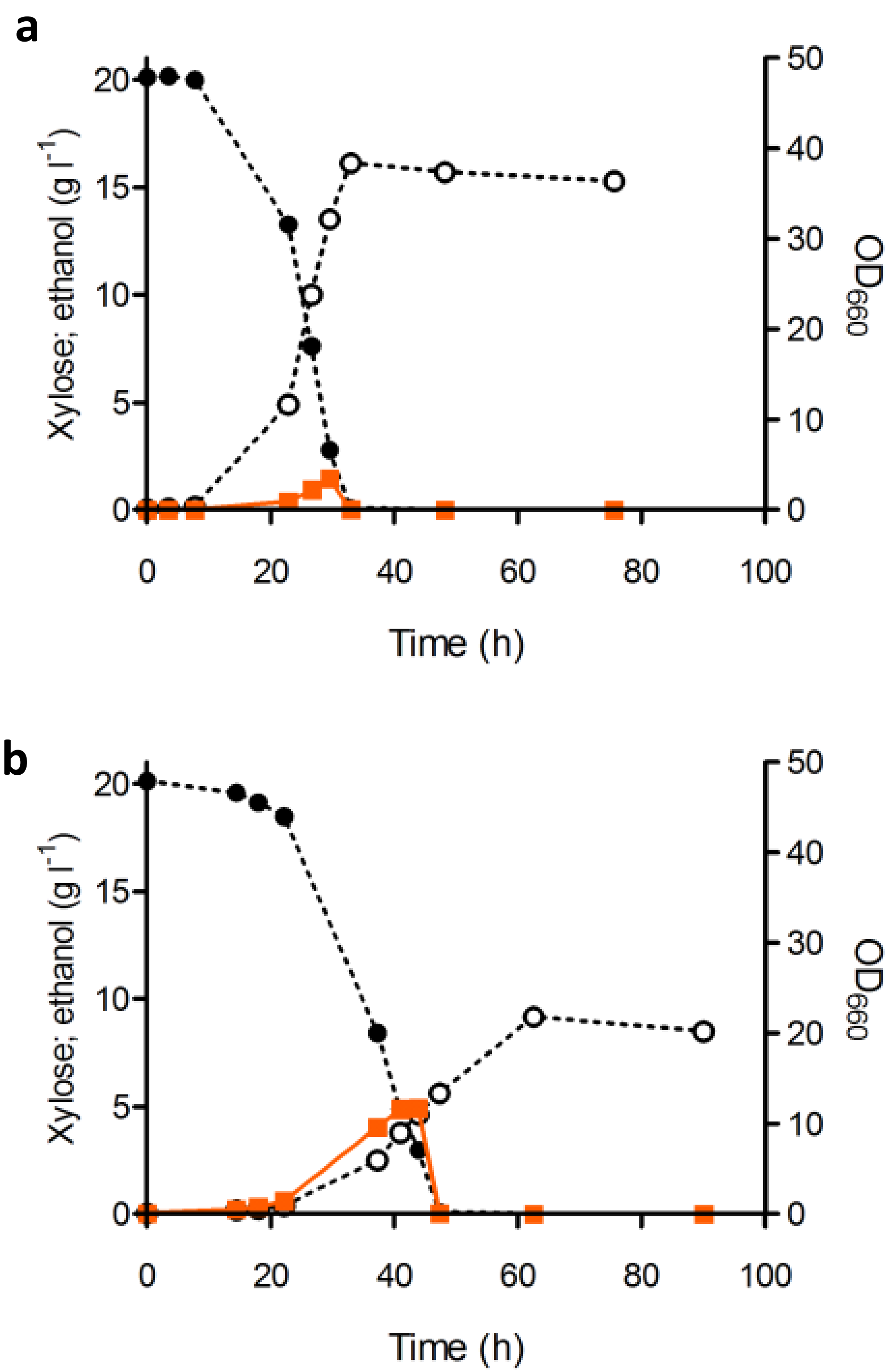

Figure S3 │ Growth of *S. cerevisiae* strains on xylose in aerobic shake-flask cultures; (**a**) IMX696 (*xylA*, PPP↑, *XKS1*↑). (**b**) IMS0488 (isolated from culture of strain IMX696 adapted to anaerobic growth on xylose). Both strains were grown in aerobic shake flasks on synthetic medium containing 20 g l^{-1} xylose. Symbols: ●, xylose, ■, ethanol and ○, biomass. The data shown in the figure are from a single shake-flask experiment of each strain. Data from duplicate experiments with each strain differed by less than 5%.

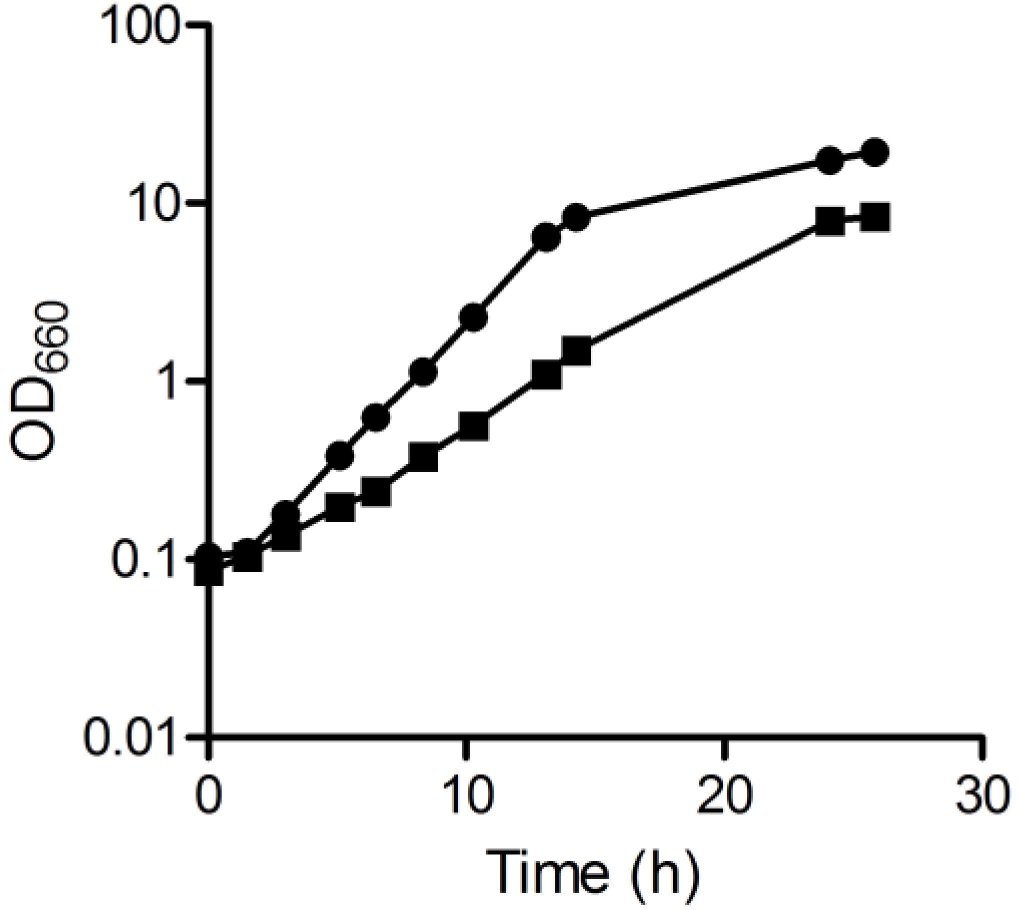

Figure S4 │ Impact of a *PMR1* deletion on aerobic growth of *S. cerevisiae* CEN.PK113-7D on glucose. Growth was monitored in aerobic shake-flask cultures grown on synthetic medium with 20 g l^{-1} glucose. Symbols indicate the following *S. cerevisiae* strains: ●, CEN.PK113-7D and ■, IMK692 (*pmr1*Δ). Data shown are from a single flask experiment for each strain. For both strains, data obtained from independent duplicate experiments differed by less than 5%.

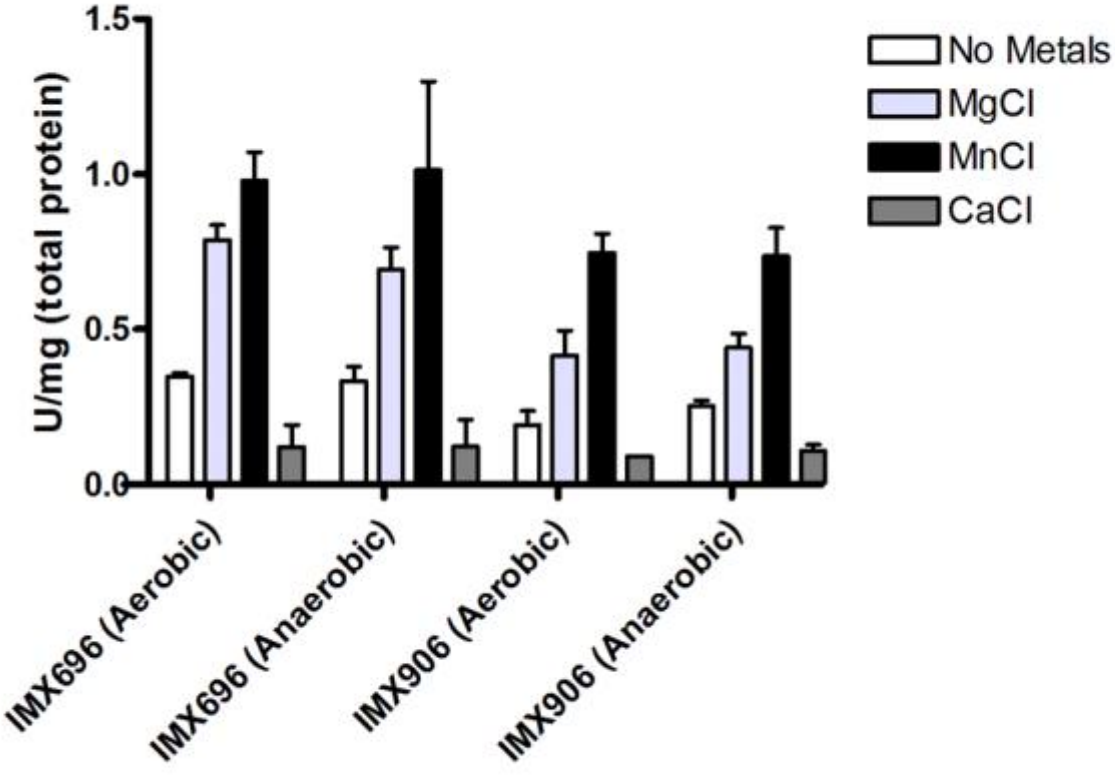

Figure S5 │XylA activity measured in cell extracts and the effect of divalent metals. XI activity measured in cell-free extracts prepared from exponentially growing shake-flask cultures of *S. cerevisiae* strains IMX696 (*xylA*, PPP↑, *XKS1*↑) and IMX906 (*xylA*, PPP↑, *XKS1*↑, *pmr1*Δ), pre-grown under aerobic and anaerobic conditions on glucose. 25-50 µl of the extract was used in a 1 ml reaction mixture containing 20 mM MOPS buffer pH 7.0, 0.25 mM NADH, 500 mM xylose, 2U sorbitol dehydrogenase and 10mM of the divalent metal ion indicated. The activity values represent average and mean deviation of independent duplicate experiments.

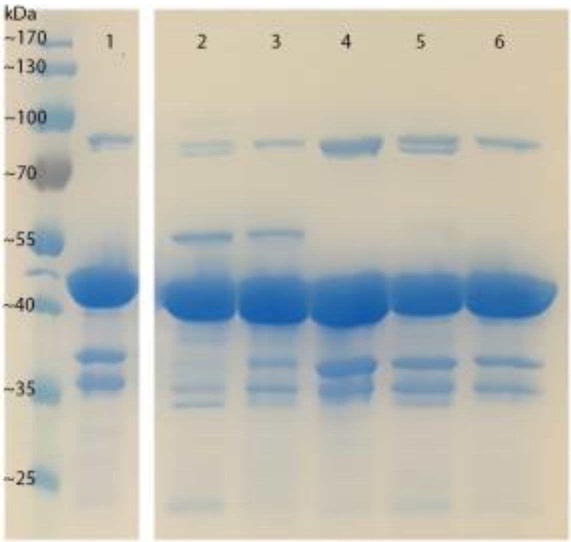

Figure S6 │ SDS-polyacrylamide gel electrophoresis of xylose isomerases isolated from different engineered *S. cerevisiae* strains. Lane 1: IMX906 ((*xylA*, *PPP*↑, *XKS1*↑, *pmr1*Δ) grown on glucose; Lane 2: IMX696 (*xylA*, *PPP*↑, *XKS1*↑) grown on glucose; Lane 3: IMX906 grown on xylose; Lane 4: IMX696 grown on glucose; Lane 5: IMX696 grown on glucose; Lane 6: IMX906 grown on xylose.

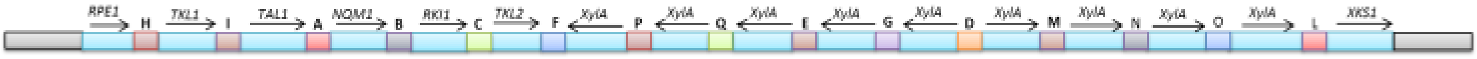

Figure S7 │ Schematic overview of the integrated construct that enables xylose consumption in IMX696 (*xylA*, PPP↑, *XKS1*↑). The construct consists of 15 cassettes containing 60bp homologous sequences named A to Q. The fragments were transformed with pUD335 allowing for a Cas9-induced double-strand break in *GRE3*. Correct integration of all the fragments in *GRE3* was verified by diagnostic PCR.
